# Using *Drosophila* Nephrocytes to Understand the Formation and Maintenance of the Podocyte Slit Diaphragm

**DOI:** 10.3389/fcell.2022.837828

**Published:** 2022-02-21

**Authors:** Joyce van de Leemput, Pei Wen, Zhe Han

**Affiliations:** ^1^ Center for Precision Disease Modeling, Department of Medicine, University of Maryland School of Medicine, Baltimore, MD, United States; ^2^ Division of Endocrinology, Diabetes and Nutrition, Department of Medicine, University of Maryland School of Medicine, Baltimore, MD, United States

**Keywords:** kidney, mammalian podocytes, Drosophila nephrocytes, slit diaphragm, apical-basal polarity, cytoskeleton, endocytosis and exocytosis, glomerular diseases

## Abstract

The podocyte slit diaphragm (SD) is an essential component of the glomerular filtration barrier and its disruption is a common cause of proteinuria and many types of kidney disease. Therefore, better understanding of the pathways and proteins that play key roles in SD formation and maintenance has been of great interest. Podocyte and SD biology have been mainly studied using mouse and other vertebrate models. However, vertebrates are limited by inherent properties and technically challenging *in vivo* access to the podocytes. *Drosophila* is a relatively new alternative model system but it has already made great strides. Past the initial obvious differences, mammalian podocytes and fly nephrocytes are remarkably similar at the genetic, molecular and functional levels. This review discusses SD formation and maintenance, and their dependence on cell polarity, the cytoskeleton, and endo- and exocytosis, as learned from studies in fly nephrocytes and mammalian podocytes. In addition, it reflects on the remaining gaps in our knowledge, the physiological implications for glomerular diseases and how we can leverage the advantages *Drosophila* has to offer to further our understanding.

## Introduction

**FIGURE 1:**
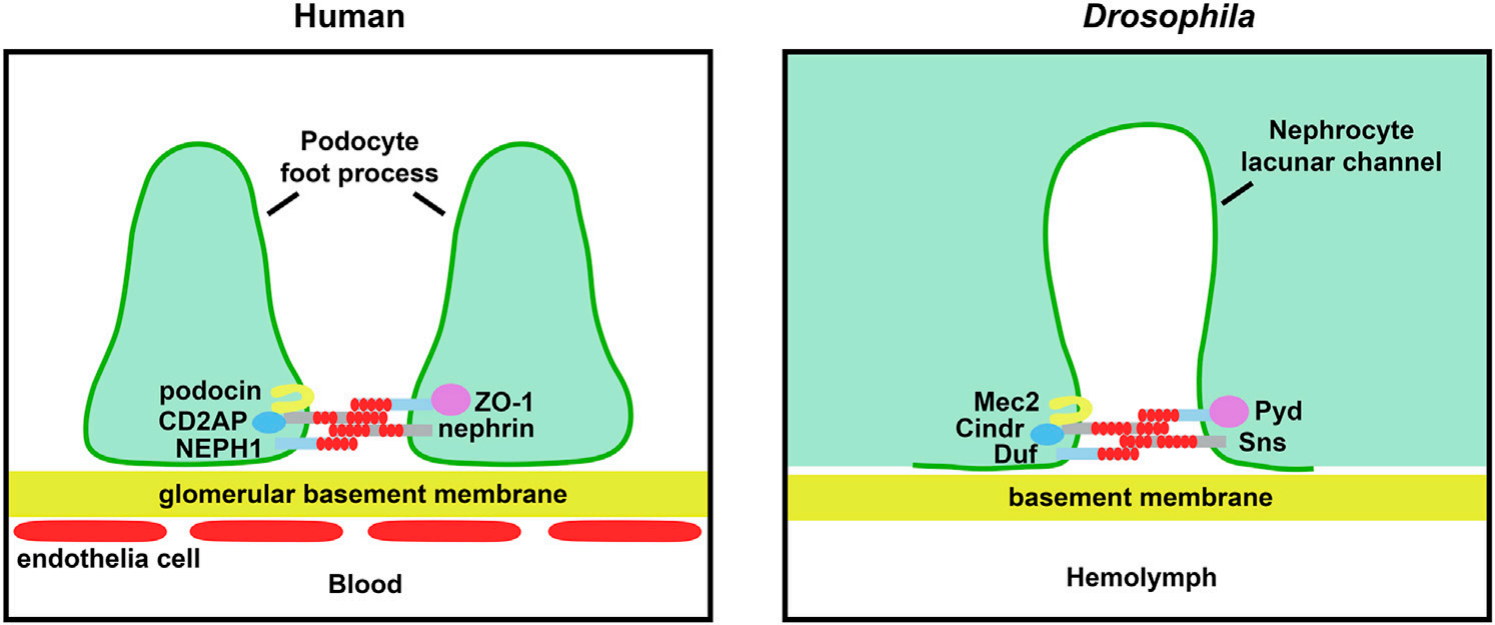
Schematic of renal filtration structure and essential slit diaphragm proteins. The left panel shows the glomerular filtration barrier structure in human. The blood is filtered through fenestrated endothelial cells, glomerular basement membrane and slit diaphragm between the podocyte foot processes. The right panel shows the filtration structure in the *Drosophila* nephrocyte. The hemolymph is filtered through the basement membrane and slit diaphragm located at the opening of the lacunar channels. The core components of the slit diaphragm are highly conserved between fly and human.

Glomerulopathy and chronic kidney disease are marked by injury and loss of podocytes. Podocytes are specialized epithelial cells that wrap around the glomerular capillaries. The podocytes tightly adhere to the outer capillary surface where they form interlaced foot processes, thus making close contacts with neighboring podocytes to form the slit diaphragms (SDs). The SDs, together with the capillary fenestrated endothelial cells and the glomerular basement membrane (GBM), form the glomerular filtration unit (Pollak et al., 2014; Kawasaki et al., 2019) (Figure 1). The filtration unit is essential for kidney function to remove toxins and waste from the bloodstream while recycling vital nutrients. These processes to maintain homeostasis of fluids (e.g. blood pressure), salts and hormones are highly conserved from Metazoa to mammals. Under stress or toxic conditions, the podocytes undergo active morphological changes during which the cells smoothen and loose their elaborate branches in a process known as foot process effacement (Garg, 2018). Because podocyte inter-foot connections are crucial to SD structural integrity, effacement inherently leads to disruption of the glomerular filtration structure and ultimately loss of kidney function. Mammalian model systems for the podocyte slit diaphragm, both *in vitro* and *in vivo*, have greatly increased our understanding. However, each system came with its own strengths and limitations. For example, the *in vitro* system provides ready access to podocytes, but their key characteristics are often rapidly lost under cell culture conditions (Agarwal et al., 2020). On the other hand, while the *in vivo* system studies podocytes in their physiological environment, the cells have been notoriously difficult to access for *in vivo* imaging (Siegerist et al., 2018). The fruit fly (i.e. vinegar flies; *Drosophila melanogaster*) is a relatively new model to the field. Here we describe the similarities and differences between the fly nephrocyte and the mammalian (mouse) podocyte filtration structures, as well as the contributions made by fly studies of SD formation and maintenance. Further, we discuss what these findings mean for human glomerular disease research, and ultimately patients, and speculate on how the unmatched fly toolkit could be employed to answer remaining questions.

## Mammalian Model Systems to Study Podocytes and the Slit Diaphragm

Mammalian models, like mouse and rat, have taught us a lot about podocytes and kidney function. Yet, intrinsic (long gestation, small litter size, high maintenance costs and limited specialized genetic tools) and experimental (difficult access to the podocytes) limitations have impeded progress. *In vitro* studies of podocytes have met their own technical challenges pertaining to the difficulty to maintain fully differentiated cells. Podocytes in culture tend to lose their most characteristic features within days, including cell polarity, the intricate foot process morphology, and intact functional SD structures. A landmark publication (Mundel et al., 1997) demonstrated that by omitting sub-cultivation of primary rat or human podocytes, columnar cells could differentiate into mature podocytes. Podocytes were designated mature based on the formation of extensive arborized processes and the expression of Synaptopodin [previously Podocyte protein, 44kDa (PP44) (Mundel et al., 1997b) and Desmin; neither protein was detected in cells with columnar morphology. However, podocyte differentiation also induced cell cycle arrest, thus limiting the number of cells available for subsequent assays (Mundel et al., 1997a). This led some researchers to develop immortalized podocyte cell lines, which came with their own set of limitations (Agarwal et al., 2020). The immortalized lines are generated through insertion of an immortalizing gene [*simian virus 40 (SV40) large tumor antigen (Tag)*] that is not naturally present in the podocyte, therefore random insertion of this gene can lead to off-target effects. The uncontrolled proliferation led to altered podocyte physiology, to prevent this conditional immortalized human podocyte cell lines using a temperature sensitive SV40 gene (*SV40-T*) have been generated. The conditional lines express Nephrin and Podocin, and using this approach podocyte lines have been derived from patients with congenital genetic syndrome (Saleem et al., 2002). These conditional lines have to be continuously monitored for spontaneous transformation, especially in podocytes cultures exceeding 30 passages (Saleem et al., 2002; Agarwal et al., 2020). Other *in vitro* disadvantages might be remedied by using more complex culture systems that mimic the circulatory system and other components of the glomerular filtration unit to better resemble physiological conditions as in, for example, the podocyte organoid models currently under development (Nishinakamura, 2019). By culturing under flow conditions, some kidney organoids include glomerular vascular development (Homan et al., 2019), other models are capable of producing functional renin, the first and rate-limiting enzyme of the renin-angiotensin system (RAS) which regulates blood pressure (Shankar et al., 2021). These technical feats require high levels of expertise and are time, cost, and labor intensive, thus limiting their widespread application. The technical difficulties in obtaining *in vivo* access to podocytes in mammalian models, and in maintaining mature podocytes *in vitro*, have spurred the development of alternative models like zebrafish (*Danio rerio*) and fruit flies (*Drosophila*).

## Conservation of the Slit Diaphragm Between *Drosophila* and Mammals

The fly equivalent of podocytes are the nephrocytes. In fact, the fly has two types of nephrocytes, those located around the connection site of the esophagus and proventriculus known as garland cells, and those located near the heart known as pericardial nephrocytes (Denholm and Skaer, 2009). Of note, unlike podocytes, the nephrocytes are not physically connected to the fly excretory and osmoregulatory system known as the Malpighian tubules (mammalian liver and kidneys). This difference means that mammalian podocytes form an *inter*cellular filtration system, between adjacent foot processes. These foot processes cover the glomerular basement membrane, which acts as a negatively charged filter prior to SD access (Haraldsson et al., 2008). At the SD, blood proteins are filtered by size for entry into the urinary space where the proteins get reabsorbed in the renal proximal tubule to retain vital nutrients. Fly nephrocytes, on the other hand, have a cell surface covered by membrane ingressions that form labyrinthine channels with a fingerprint-like pattern. *Auto*cellular filtration barriers span the openings of these labyrinthine, or lacunar, channels where hemolymph proteins get reabsorbed *via* endocytosis (Kawasaki et al., 2019) (Figure 1). The nephrocytes are encased in the basement membrane (Kawasaki et al., 2019), where it might perform a similar function to the podocyte glomerular basement membrane. Unfortunately, the nephrocyte basement membrane remains a grossly understudied area. Given the close interaction between the SD and the basement membrane and their size-charge filtration activities, respectively, further study of the basement membrane will undoubtedly benefit our understanding of the SD in development and diseases.

The fly might not have a defined kidney as such, but the pathways governing filtration of its hemolymph (fly equivalent of blood) are remarkably conserved among flies and mammals. Insect nephrocytes have been described as early as the 1800s. Initially they were identified as phagocytic cells able to take up foreign and toxic compounds from the hemolymph (Leydig, 1866; Balbiani, 1886; Hollande, 1921; Crossley, 1972). However, it was not until landmark publications in 2009 (Weavers et al., 2009; Zhuang et al., 2009) that their similarity to mammalian podocytes was fully appreciated and their potential as a model system recognized. These studies showed that both podocytes and nephrocytes have a specialized size-selective filtration barrier, and that the fly nephrocyte has orthologs for the main mammalian proteins composing the SD, including fly Sns (mammalian Nephrin), Duf [Kin of irre (kirre)] (mammalian KIRREL1; NEPH1), Cindr (mammalian CD2AP), Pyd (mammalian ZO-1), and Mec2 (mammalian Podocin). The studies also demonstrated that these proteins form similar multi-protein complexes in both species, that altered expression of these complexes leads to defective filtration and disrupts hemolymph homeostasis, and that Sticks and stones (Sns) and Dumbfounded (Duf), similar to their mammalian Nephrin and Nephrin-like 1 (NEPH1) counterparts, are key to SD formation (Weavers et al., 2009; Zhuang et al., 2009). Together these studie demonstrated the strong similarities between the fly nephrocyte and mammalian podocyte SD structures. Moreover, the width of the SD structure in podocytes and nephrocytes is comparable at just under 40 nm (Kawasaki et al., 2019), and podocytes and pericardial nephrocytes share a ∼70 kD size selection filtration cut-off (garland nephrocytes filtrate at a 66–80 kD size limit) (Hermle et al., 2017). The highly conserved genes, pathways, filtration structure and function, combined with the highly accessible nephrocytes (facilitated by the open circulatory system in fly, whereas podocytes are locked away inside glomeruli), have made the fly a powerful model to study podocyte development and disease.

## Role for Core Components of the Slit Diaphragm in Its Assembly

Zonula occludens-1 (ZO-1) and NEPH1 are two core components of the SD main filtration unit (Figure 1 and Table 1). It has been shown these proteins themselves play critical roles in junctional remodeling and formation of the SD (Carrasco-Rando et al., 2019). Nephrocytes deficient for Sns or Duf, fly orthologs of mammalian Nephrin and NEPH1 respectively, show a near complete lack of SD structures at the labyrinthine lacunar channels and display a significantly impaired filtration function (Weavers et al., 2009; Zhuang et al., 2009). In addition, ZO-1 localization to the podocyte membrane is dependent on NEPH1-ZO-1 complex formation (Wagner et al., 2008). Similarly, in fly nephrocytes localization of Duf is dependent on Pyd (mammalian ZO-1) during formation of the lacunar channel filtration unit (Carrasco-Rando et al., 2019). For both mammalian podocytes and fly nephrocytes this transition from junctional complex (Cadherin-based) to SDs is reversible (Carrasco-Rando et al., 2019). In fact, the reversed process underlies foot process effacement which is a common hallmark of glomerular diseases. In podocytes, CD2-associated protein (CD2AP) direct interaction with Nephrin and Podocin was shown to be important for SD structural integrity (Schwarz et al., 2001; Shih et al., 2001). Similarly, a genetic interaction study in nephrocytes, showed that the fly ortholog CIN85 and CD2AP related (Cindr) (mammalian CD2AP) interacts with Mechanosensory protein 2 (Mec2) (mammalian Podocin) Fly nephrocytes deficient for Cindr showed collapsed lacunar channels and effacement of SDs, culminating in severe functional defects, and reduced lifespan (Fu et al., 2017b). Nephrocytes from flies on a chronic high sucrose diet, a model for diabetic nephropathy, showed reduced Sns. Further, the study showed that the transcription factor Knot (Kn) [mammalian Early B cell factor 2 (EBF2)], negatively regulated Sns expression in fly nephrocytes in a high dietary sugar dependent manner. The same negative correlation was found for EBF2 in regulating the level of Nephrin in glomeruli of a patient with diabetic nephropathy (Na et al., 2015). Together these findings indicate an important role for these core filtration proteins in SD formation and possibly maintenance (Figure 1).

**TABLE 1:** Essential slit diaphragm proteins.

Human protein	Fly ortholog	DIOPT score	Protein function	Function in podocytes/nephrocytes
Nephrin	Sns	14	Cell junction	SD Component
NEPH1	Duf	11	Cell junction	SD Component
ZO-1	Pyd	11	Cell junction	SD Component
CD2AP	Cindr	8	Cell junction	SD Component
Podocin	Mec2	3	Cell junction	SD Component
PRKCI (aPKC)	aPKC	12	Cell polarity	SD Formation
PARD6 (G/B/A)	Par-6	12	Cell polarity	SD Formation
PARD3	Baz	13	Cell polarity	SD Formation
DLG1	Dlg	13	Cell polarity	SD Formation
LLGL1	Lgl	15	Cell polarity	SD Formation
SCRIB	Scrib	7	Cell polarity	SD Formation
MARK1	Par-1	10	Cell polarity	SD Formation
STK11 (LKB1)	Lkb1	12	Cell polarity	SD Formation
CRB1	Crb	9	Cell polarity	SD Formation
PALS1 (MPP5)	Sdt	12	Cell polarity	SD Formation
PATJ	Patj	9	Cell polarity	SD Formation
EZR/RDX/MSN	Moe	13/13/14	Actin regulation	Cytoskeleton
ARHGDIA	RhoGDI	15	Actin regulation	Cytoskeleton
ARHGAP24	RhoGAP92B	1	Actin regulation	Cytoskeleton
KANK1, 2, 4	Kank	10	Actin regulation	Cytoskeleton
MYH9	Zip	11	Actin regulation	Cytoskeleton
ACTN4	Actn	11	Actin regulation	Cytoskeleton
MYO1E	Myo61F	3	Actin regulation	Cytoskeleton
INF2	Form3	7	Actin regulation	Cytoskeleton
CLTA	Chc	13	Endocytosis	SD Maintenance
CLTC	Clc	14	Endocytosis	SD Maintenance
DYNAMIN	Shi	13	Endocytosis	SD Maintenance
AP2B1	AP-1-2β	12	Endocytosis	SD Maintenance
AP2A2	AP-2α	13	Endocytosis	SD Maintenance
AP2M1	AP-2μ	13	Endocytosis	SD Maintenance
AP2S1	AP-2σ	14	Endocytosis	SD Maintenance
AUXILIN	Aux	11	Endocytosis	SD Maintenance
HSPA8	Hsc70-4	12	Endocytosis	SD Maintenance
PICALM (AP180)	Lap	12	Endocytosis	SD Maintenance
RAB5B	Rab5	13	Endocytosis	SD Maintenance
RBSN	Rbsn-5	11	Endocytosis	SD Maintenance
PIK3C3 (VPS34)	Pi3K59F (Vps34)	15	Endocytosis	SD Maintenance
GAPVD1	Gapvd1	14	Endocytosis	SD Maintenance
AMN	dAmn	11	Endocytosis	SD Maintenance
CUBN	Cubn	12	Endocytosis	SD Maintenance
CUBN	Cubn-2	9	Endocytosis	SD Maintenance
RPH3a	Rph	10	Endocytosis	SD Maintenance
RAB3A	Rab3	12	Endocytosis	SD Maintenance
RAB11A	Rab11	13	Recycling	SD Maintenance
EXOC1	Sec3	14	Recycling	SD Maintenance
EXOC2	Sec5	14	Recycling	SD Maintenance
EXOC3	Sec6	14	Recycling	SD Maintenance
EXOC4	Sec8	14	Recycling	SD Maintenance
EXOC5	Sec10	14	Recycling	SD Maintenance
EXOC6	Sec15	13	Recycling	SD Maintenance
EXOC7	Exo70	15	Recycling	SD Maintenance
EXOC8	Exo84	14	Recycling	SD Maintenance
TBC1D8B	Tbc1d8b	13	Recycling	SD Maintenance

DIOPT, *Drosophila* RNAi Screening Center (DRSC) integrative ortholog prediction tool (Hu et al., 2011); SD, slit diaphragm.

## The Importance of Apical-Basal Polarity for Slit Diaphragm Formation

**FIGURE 2:**
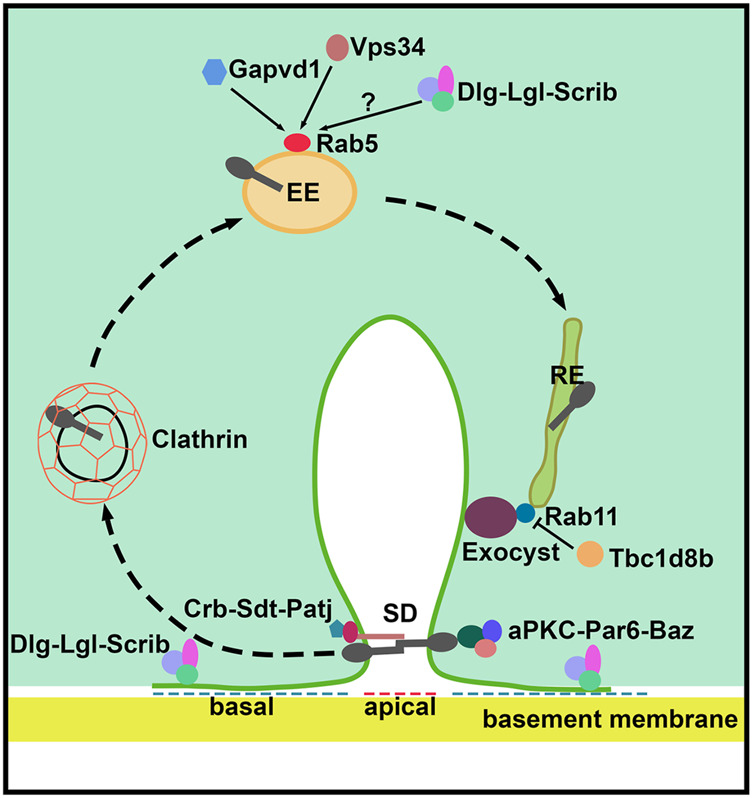
Schematic of slit diaphragm formation and maintenance in *Drosophila* nephrocytes. The apical polarity Crumbs (Crb) and aPKC complexes are localized at the slit diaphragm and define the apical region of the nephrocyte which is important during slit diaphragm formation. The basolateral Scribble (Scrib) complex localizes to the basal region of the nephrocyte between neighboring slit diaphragms. The clathrin-mediated endocytosis, Rab5-dependent early endosomes, Rab11-dependent recycling endosomes and the exocyst complex are critical for slit diaphragm maintenance. Gapvd1, Vps34 (Pi3K59F), and possibly the Scrib basal polarity complex, positively regulate Rab5-dependent early endosome trafficking. Tbc1d8b negatively regulates the function of Rab11 in slit diaphragm recycling. SD, slit diaphragm; EE, early endosome; and, RE, recycling endosome.

Podocytes have an epithelial origin, starting out as cells with columnar morphology that support cell-cell junctions at the apical surface. These epithelial cells completely transform during development, turning into podocytes with bulbous cell bodies and intricate protrusions that form intercellular junctions (i.e. SDs) at their basal foot processes. This transformation requires (re-)localization of many apical junction proteins to the basal side of the podocyte, where they are gradually replaced by the SD (Ichimura et al., 2017). Even though nephrocytes are not derived from epithelial progenitor cells, they do show the distinct apical-basal polarity (Heiden et al., 2021). The apical and basal plasma membrane domains are defined by the junctional zone and SD/lacunar channel diaphragms in mammalian podocytes and fly nephrocytes, respectively. Moreover, apical-basal polarity in both cell types is governed by the same classical polarity proteins, which have been found to play key roles in SD assembly, maintenance and endocytosis (Michgehl et al., 2017; Heiden et al., 2021) (Figure 2 and Table 1).

### The Role of Apical Polarity Proteins

The apical polarity Crumbs (CRB) and Partitioning defective (PAR)-atypical Protein kinase C (aPKC) complexes have been implicated in podocyte health, and SD formation and maintenance. The polarity protein known as Protein crumbs homolog (CRB) is an evolutionary highly conserved protein that is important during epithelial morphogenesis. The genes encoding components of the CRB complex, including fly Crumbs (gene *crb*), Stardust (gene *sdt*) and Bazooka (gene *baz*), were initially discovered in a systematic screen for fly developmental mutants (Wieschaus and Nüsslein-Volhard, 2016). The genes encoding the CRB complex proteins were noted for their disruptive effect on epidermal structure and integrity when mutated. The fly and mammalian CRB complexes share core components, including Sdt [mammalian Protein associated with LIN-7 1 (PALS1)], Serine/threonine-protein kinase Par-6 (Par-6), and Pals1-associated tight junction protein (Patj) (Hochapfel et al., 2017; Michgehl et al., 2017). Fly nephrocytes deficient for *crb* displayed reduced SD structures, disturbed morphology of foot processes and a marked decreased filtration rate (*ex vivo*). This could be attenuated by expressing either human *CRB2* or *CRB3*, which encode the two CRB proteins expressed in podocytes (Hochapfel et al., 2017). Mammalian PALS1 (a.k.a. MPP5) is a core component of the CRB complex in the nephron. Mice with nephron-specific *Pals1* haploinsufficiency suffered from renal cyst formation and severe defects in renal barrier function, indicative of abnormal SDs, resulting in early death (6–8 weeks) (Weide et al., 2017). Similarly, the PALS1 fly homologous protein Sdt was found to be essential in regulating epithelial cell polarity and for SD structural formation in nephrocytes (Bachmann et al., 2001; Hong et al., 2001). Deficiency for *sdt* led to destabilization and endocytosis of Crb in fly epithelial cells (Lin et al., 2015), with mis-localization of Crb (and Patj) to the cytosol and vesicles, and strikingly decreased expression in the nephrocytes, while Sns remained at the cortex (Hochapfel et al., 2017). Moreover, Crb regulates early- and late-endosome formation in nephrocytes, indicating a role in SD maintenance and function (Hochapfel et al., 2017). Regarding the PAR polarity complex, studies in mouse podocytes have shown PAR complex components PAR3, PAR6 and aPKC interact with NEPH1-Nephrin at the SD, and that this interaction is required to establish the structures for the foot processes and SD filtration barriers which facilitate glomerular maturation (Hartleben et al., 2008, 2013). Even though aPKC λ/ι and aPKC ζ isoforms are very similar in structure, both are needed to stabilize the NEPH1-Nephrin complex at the SD (Hartleben et al., 2013). Recently, *in vivo* studies have shown similar processes are active in fly nephrocytes. The studies demonstrated that fly Par-6, aPKC and Baz (mammalian PAR-3) are essential for localization of Sns (mammalian Nephrin) at the nephrocyte cortex (Heiden et al., 2021). In addition, the Par-aPKC complex has been shown to be important in endocytosis and SD structural integrity in nephrocytes (Heiden et al., 2021). Together, these studies suggest a role for PAR-aPKC in SD maintenance and function as well as formation.

### The Role of Basolateral Polarity Proteins

In addition to the apical polarity regulators, basolateral polarity determinants were found to be essential for slit diaphragm formation. The actions of the apical CRB and PAR-aPKC complexes are balanced by the basolateral Scribble complex. The Scribble complex, comprising Discs large (Dlg), Scribble (Scrib), and Lethal giant larvae (Lgl), was first discovered in *Drosophila* (Hadorn, 1937; Scharrer and Hadorn, 1938; Stewart et al., 1972; Bilder and Perrimon, 2000), with subsequent uncovering of their mammalian orthologs [*see* (Su et al., 2012) for a detailed review]. The balancing actions of the apical and basal complexes was recently demonstrated in fly nephrocytes, which showed that the basolateral polarity proteins Dlg, Scrib, Lgl, and Par1 are required for SD formation (Mysh and Poulton, 2021). Subcellular localization and genetic-interaction data suggested interaction between basolateral and apical protein functions. However, the authors caution that it remains to be determined whether these effects are due to direct interaction of the basolateral and apical protein complexes or if they are mediated through their interaction with the SD (Mysh and Poulton, 2021). Furthermore, the study showed that nephrocytes deficient for the basolateral polarity proteins, particularly Dlg and Par1, displayed mis-localized SD proteins. These nephrocytes showed fewer lacunar channels and SD structures. Instead, the SD components were located in cytoplasmic vesicles adjacent to Rab5-positive vesicles (early endosomes). Based on these findings the authors proposed that the basolateral proteins mediate the organization and function of the endocytic pathway (Mysh and Poulton, 2021). The importance of endocytosis and various Rab family members, including Rab5, in maintaining SD structural integrity—by ensuring localization of key SD proteins—and supporting nephrocyte function has been recently reported in fly (Wen et al., 2020). Furthermore, a separate study in nephrocytes, which used a combination of expansion microscopy and functional assays, showed that the basolateral polarity regulators Dlg, Lgl, Lkb1, and to a greater extent Par-1 and Scrib, are essential for proper Sns distribution (Heiden et al., 2021). Deficiency for any of these polarity proteins led to reduced presence of Sns at the cell surface, with Sns re-localized to intracellular vesicles. Despite the greatly reduced SDs in these nephrocytes—observed as a severely disturbed Sns distribution on the nephrocyte cell surface that typically presents as a fingerprint-like pattern—uptake seemed mostly intact in functional assays of endocytosis (Heiden et al., 2021). Notably, mouse podocytes deficient for *Scrib* (Hartleben et al., 2012) or *Dlg5* (Yu et al., 2016) did not disrupt cell polarity, SD structural integrity or podocyte function (no detectable proteinuria). A plausible explanation [suggested by (Mysh and Poulton, 2021)] for the lacking effect in mice is the genetic redundancy for basolateral polarity components observed in mouse and human, which suggests protein family members might compensate for any deficiency. Indeed, Scrib belongs to the LAP family with four family members [SCRIB, Leucine-rich repeat-containing protein 1 (LRRC1), LRRC7, and Erbb2-interacting protein (Erbin)], and the DLG protein family contains five members (DLG1-5) in mammals. The fly genome, on the other hand, is overall much more compact with little genetic redundancy. It comprises two LAP orthologs (Scrib, and Lap1) and two Dlg protein family members (Dlg1 and Dlg5) (https://flybase.org; http://www.flyrnai.org/diopt). To determine whether these components of basal polarity play similar roles in mammalian podocytes as their roles in fly nephrocytes will require further investigation that combines multiple family members within one model. Interestingly, the apical polarity complexes, CRB and PAR-aPKC, similarly mediate endocytosis (Hochapfel et al., 2017; Heiden et al., 2021), suggesting intersection of the basal (Scrib, Dlg) and apical (Crb, Par-aPKC) polarity pathways. Additional studies are needed to determine how these apical-basal polarity components interact with the SD structural proteins and other podocyte/nephrocyte functional components in the pathomechanisms underlying glomerular diseases.

## Slit Diaphragm Dependence on the Cytoskeleton for Formation and Function

The role of the cytoskeleton in podocyte development and function cannot be overstated. During development the podocytes undergo huge morphological changes, transforming from simple columnar epithelial cells into the fully differentiated podocytes marked by a bulbous cell body and complex network of protrusions (foot processes). In addition to structure and shape, the podocyte cytoskeleton is also integral to forming the refined polar organization required to obtain the intricate arborization of mature podocytes. Furthermore, the cytoskeleton plays a crucial role in podocyte functioning by ensuring adhesion of the foot processes to the basement membrane, which is essential for the formation of the main filtration structures (slit diaphragms), as well as for adequate signaling responses to external stimuli (Welsh and Saleem, 2011; Endlich et al., 2017; Blaine and Dylewski, 2020). To achieve its complex morphology, each podocyte cellular compartment carries a distinct combination of cytoskeletal components. The major extending, primary processes, that protrude from the bulbous cell body, contain a microtubule and intermediate filament cytoskeleton, while the finer branches, known as secondary foot processes, are mainly structured by Actin filaments (Drenckhahn and Franke, 1988; Schell and Huber, 2017).

### The Cytoskeleton and the Slit Diaphragm

Core SD components Duf and Sns are members of the immunoglobulin superfamily that is known to mediate cell adhesion, among others, and to influence cytoskeletal organization through adaptor proteins. In nephrocytes, it has been shown that Pyd-P, a specific isoform of Pyd, might serve as such an intracellular Actin-associated adaptor protein for Duf, this interaction mediates Duf-Sns assembly at the SD. Indeed Pyd is a component of both adherens junctions and the SD filtration barrier, this dual function might facilitate the recruitement and organization of the nephrocyte cytoskeleton (Carrasco-Rando et al., 2019). Similarly, in podocytes disruption of the NEPH1-ZO-1 complex (fly Duf-Pyd) leads to mis-localized NEPH1 and its reduced presence at the SD (Wagner et al., 2008). Moesin is a member of the Ezrin, Radixin, Moesin (ERM) protein family. These proteins interact with transmembrane proteins and membrane associated cytoplasmic proteins on one side, and the filamentous Actin cytoskeleton on the other (Tsukita and Yonemura, 1999). Ezrin is highly expressed in podocytes, where it has been reported to act in several complexes, including with the Na^+^/H^+^ exchanger regulatory factor (NHEFR2) and Podocalyxin. The function of Ezrin in podocytes might be multi-fold. Interestingly, a study in mice showed *Ezrin* deficiency (knockdown) possibly protects podocytes from morphological defects by inhibiting Rac1 activation (Hatano et al., 2018). The fly homolog for all three ERM family proteins is Moesin (Moe). Nephrocytes deficient for *Moe* (RNAi) displayed a disrupted Actin cytoskeleton and SD structures (Hochapfel et al., 2017). Furthermore, these nephrocytes showed reduced early (Rab5-positive) and, to lesser extent, late (Rab7-positive) endosomal vesicles. A phenotype reminiscent of nephrocytes deficient for *crb* (RNAi) in the same study. In fact, *Moe*-deficient nephrocytes showed decreased cortical Crb expression, and *vice versa*. Moreover, the phenotype in *crb*-deficient nephrocytes could be attenuated by simultaneous overexpression of *Moe*. These interdependencies were shown to be contingent on the intracellular FERM-binding domain of Crb (Wei et al., 2015; Hochapfel et al., 2017). Fly and mouse studies have demonstrated Crb is competitively regulated by Moesin (Actin cytoskeleton) and aPKC (apical polarity) (Whiteman et al., 2014; Sherrard and Fehon, 2015). Further studies are needed to identify the molecular pathways underlying Crb and Moesin-mediated endocytosis, and their possible ties to mechanisms of cellular polarity. Another example of the involvement of the cytoskeleton at the SD is the functional interactions of Dynamin (a GTPase), Synaptojanin and Endophilin. These proteins are known for their role in synaptic vesicle recycling in neurons, where they are critical for membrane fission during Clathrin-dependent endocytosis and vesicular trafficking. Their function is Actin-dependent, and their regulatory complexes and pathways are highly conserved from yeast to mammals (Kaksonen and Roux, 2018). A study in mice podocytes showed that Dynamin, Synaptojanin and Endophilin, similar to neurons, act at the intersection of endocytosis and the Actin cytoskeleton, and are critical for SD formation. Mice deficient for any of these proteins displayed substantial proteinuria at birth. They further found reduced turnover of Nephrin (fly Sns) on the surface of podocyte foot processes (Soda et al., 2012). Subsequently, Dynamin-1 was shown to regulate the formation and maintenance of the microtubule network in rat glomerular podocytes (La et al., 2020). These Dynamin protein interactions in podocytes are highly conserved. In flies, Dynamin is known as Shibire (Shi), which was similarly shown to be required for SD protein endocytosis in nephrocytes (Wang et al., 2021). Given the high conservation of these endocytosis and vesicular trafficking pathways, the fly nephrocyte could provide a valuable model for further studies.

### Systematic Studies Into Podocyte/Nephrocyte Cytoskeletal Proteins

Systematic studies to identify the proteins essential for podocyte structure and function have uncovered multiple Actin cytoskeleton-related proteins. One such study carried out an *in vitro* genetic screen based on podocyte adhesion to various substrates. Among the genes affecting adhesion were several encoding Actin regulatory proteins (Cinà et al., 2019), including: Integrin-linked protein (ILK) which mediates Actin filament rearrangement (Qian et al., 2005); Parvin alpha (PARVA), an Actin-binding protein; and, PDZ and LIM domain 2 (PDLIM2), an adaptor protein of the Actin cytoskeleton that promotes cell attachment. Another study used flies combined with RNAi and conditional CRISPR/Cas9 in nephrocytes to test 29 genes associated with steroid-resistant nephrotic syndrome (SRNS) (Hermle et al., 2017). Of these, 16, when deficient in fly nephrocytes, caused loss-of-function based on a tracer endocytosis assay. These genes encoded fly orthologs of proteins in the SD complex, proteins involved in endocytosis, and proteins that regulate the Actin cytoskeleton, among others, the latter included: orthologs for mammalian α -Actinin-4 (ACTN4), Rho GTPase-activating protein 24 (ARHGAP24), Myosin-9 (MYH9), and Anillin (ANLN). A second study in fly, systematically tested 40 genes previously associated with nephrotic syndrome, including several not studied *in vivo* before (Fu et al., 2017b). Nephrocyte-specific RNAi silencing revealed that 85% of the disease-associated genes were required for nephrocyte uptake function. These included seven that encode proteins known to regulate Actin cytoskeletal dynamics: RhoGDI [mammalian Rho GDP-dissociation inhibitor 1 (ARHGDIA)] and Kank [mammalian KN motif and ankyrin repeat domain-containing proteins 1 (KANK1)] (both previously shown), as well as Zip (mammalian MYH9), Actn (mammalian ACTN4), integrins Mew [mammalian Integrin alpha-3 (ITGA3)] and Mys [mammalian Integrin beta-4 (ITGB4)], homeobox transcription factors CG32105 [mammalian LIM homeobox transcription factor 1-beta (LMX1B); important for maintenance of the Actin cytoskeleton and regulation of the SD]. Nephrocytes deficient for these genes, displayed disrupted SDs and lacunar channel structures as well as functional deficits (Fu et al., 2017b).

These findings likely describe just a fraction of the structural and functional (e.g. protein trafficking) contributions of the cytoskeleton in podocytes/nephrocytes. Much remains to be learned, such as how disruption of the microtubule cytoskeleton affects apical-basal polarity and thus podocyte/nephrocyte maturation and formation of the SD. The nephrocyte cytoskeleton, especially the microtubule network, remains a greatly understudied area. However, the fly shares many of the proteins and interactions that form and regulate the cytoskeleton. The fly could rapidly accommodate extensive initial screens of genetic interactions with components of the cytoskeleton prior to further study in more expensive and time-consuming mammalian model systems.

## Endocytosis and Exocytosis Sustain Slit Diaphragm Dynamics and Maintenance

The SD is highly dynamic and requires rapid turnover of its core components to ensure responsive uptake of proteins from blood, and to safeguard podocyte homeostasis. Therefore, SD maintenance is heavily dependent upon endocytosis and exocytosis (Figure 2 and Table 1). The class III Phosphoinositide 3-kinase vacuolar proteins sorting 34 (Vps34; a.k.a. fly Pi3K59F; a.k.a. mammalian PIK3C3) has been found to be one such protein indispensable for SD maintenance. A study using both mouse and fly models demonstrated a key role for Vps34 in regulating endocytosis and autophagosome-autolysosome formation in podocytes and nephrocytes, respectively (Bechtel et al., 2013). In both cells, *Vps34*-deficiency resulted in severe disruption of the early (Rab5-positive) endosomal compartment. The *Vps34*-deficient fly nephrocytes revealed a disruption between Rab5 and Rab7-positive endosomal compartments, these mis-localized proteins are likely indicative of disrupted vesicular transport which ultimately leads to diminished fluid-phase uptake. The mouse podocyte data indicated endosomal deficits, rather than aberrant autophagosome formation and autophagic flux, as the primary cause of the extensive vacuolization and foot process effacement. Mice with podocytes deficient for VPS34 suffered from proteinuria, glomerular scarring, and early lethality (Bechtel et al., 2013). Of note, a class II Phosphoinositide, 3-kinase C2 α (PI3KC2 α ), was found to be essential for podocyte morphology and function in mice, as deficiency led to chronic renal failure, marked by podocyte loss (atrophy), widespread foot process effacement, and modest proteinuria (Harris et al., 2011). While these findings implicate PI3KC2 α in podocyte maintenance, it remains to be seen how the associated morphological disruption might affect SD structural integrity. The endocytosis and exocytosis pathways are highly conserved. Indeed, a systematic study of 27 *Drosophila* Rab GTPases, found nearly half were expressed in, and required for, nephrocyte function (Fu et al., 2017c). The study identified Rabs 1, 5, 7, 11, and 35 as essential. Notably these include Rabs that mediate early and late endosomes and the recycling vesicle trafficking pathways. Furthermore, *Rabphilin 3A* (*RPH3A*) which encodes a Rab small GTPase family effector protein has been implicated in SD functional integrity (Selma-Soriano et al., 2020). RPH3A forms a complex with Rab3A, which is known for its role in vesicle docking/fusion reactions during endocytosis and exocytosis at the neuronal synapse. Expression of the RPH3a-Rab3a complex was shown around vesicles located in the foot processes of mouse, rat, and human podocytes in tissue samples (Rastaldi et al., 2003). However, the effect of a RPH3a-mediated pathomechanism might have on podocytes remained unknown. A recent study in fly showed that Rph (mammalian RPH3A) colocalized with endocytic pathways proteins, Rab3 and Hepatocyte growth factor-regulate tyrosine kinase substrate (Hrs). Hrs plays a role in endocytic sorting of ubiquitinated membrane proteins (Selma-Soriano et al., 2020). Nephrocytes deficient for Rph, displayed a reduced number of endosomes with reduced uptake of toxins, and reduced expression of SD core components like Cubn and Sns. Indeed, lacunar channels and SD structures in these nephrocytes were disrupted, which led to an overall loss of nephrocytes in the flies (Selma-Soriano et al., 2020). Finally, studies have shown the importance of both endocytosis and vesicle recycling to maintain the structural integrity of the SD (Wen et al., 2020; Wang et al., 2021). The exocyst complex is an octameric protein complex important for exocytosis and recycling that tethers the vesicles to the plasma membrane thus mediating fusion. Mice deficient for *Exoc5*, one of the exocyst complex proteins, displayed severe proteinuria and glomerular defects (Nihalani et al., 2019). The components that make up the exocyst complex are highly conserved. In fact, silencing each of the genes encoding the exocyst components in fly nephrocytes demonstrated the importance of the exocyst complex for lacunar channel membrane invagination and SD structural integrity. Flies deficient for exocyst components showed mis-localization of key SD proteins and dysfunctional nephrocytes with significantly reduced protein uptake. The study further showed that SD proteins partially co-localized with exocyst components Sec15, Rab5, and Rab11 (Wen et al., 2020). Together, the data suggest that slit diaphragm proteins are endocytosed through Clathrin-mediated endocytosis, then enter Rab5-labelled early endosomes, and are sorted into Rab11-dependent recycling endosomes. Rab11 then interacts with the Sec15 subunit of the exocyst complex, which tethers the recycling endosome to the cell membrane and promotes the fusion of their respective membranes. This facilitates recycling of the slit diaphragm proteins back to the plasma membrane (Wen et al., 2020). The key proteins of this endocytosis and recycling route are known to regulate cellular trafficking. It remains unclear whether they have any direct interactions with slit diaphragm proteins or carry out podocyte-specific functions.

## Challenges in Slit Diaphragm Research and Advantages of Using *Drosophila*

To date few, if any, studies have distinguished between the proteins important for SD assembly versus those in maintenance or function. Most glomerular model studies have focused on foot process effacement, a later stage outcome, rather than SD assembly. The aPKC double mutant (aPKC λ/ι and aPKC ζ ) study in mice (Hartleben et al., 2013) was able to investigate stages prior to effacement by focusing on the late capillary loop stage, i.e. the stage of immature glomeruli where cell division is arrested and typical podocyte differentiation markers like Nephrin and Podocin are expressed. The study made a strong case for a role for aPKCs and the apicobasal polarity PAR complex in SD formation, however, the study did not investigate whether aPKC proteins play a role in SD maintenance. A separate study found that mouse podocytes deficient for aPKC λ show SD disassembly, disrupted apico-basal cell polarity, and focal segmental glomerulosclerosis (FSGS; scarring, i.e. sclerosis, of the kidney), indicating a role in SD maintenance (Hirose et al., 2009). The study further found evidence for a direct interaction between the aPKC-PAR3 complex and the core SD Nephrin-Podocin complex. Through this interaction, aPKC-PAR3 regulates Nephrin and Podocin distribution and Nephrin accumulation at the plasma membrane (Hirose et al., 2009). These studies highlight the importance of the temporal component. The timecourse of events is especially relevant in differentiating between SD formation and maintenance. The same group studied VPS34 and its role in podocyte homeostasis and related SD maintenance (Bechtel et al., 2013). Their previous work had shown that constitutive knockout of *Vps34* led to embryonic death (day 7.5–8) in mice (Zhou et al., 2010), therefore, this time they used a conditional mouse model with podocyte-specific knockdown of *Vps34*. The conditional model revealed a crucial role for VPS34-mediated endocytosis in podocyte function and SD structural integrity. This latter study exemplifies the importance of the spatial, i.e. tissue specific, component of studies into podocyte development and SD formation. These temporal and spatial aspects are technically difficult, time-consuming, and costly to study in mammalian models. Fortunately, the fly offers a well-founded alternative.

*Drosophila* offers a plethora of inducible systems for genetic manipulation, while its fast lifecycle offers the opportunity for detailed timecourse studies with reasonable effort and cost. In fact, the timing of the developmental stages in fly are so well defined that many stage-specific drivers for gene expression are readily available (*Gal4*-UAS system) (Brand and Perrimon, 1993). Alternatively, one could use temperature sensitive RNAi, regulated by simply housing the flies at different temperatures. Moreover, cell subtype-specific genetic manipulation can be achieved by crossing any of the numerous split-Gal4 fly lines in which Gal4 activity is dependent upon two enhancers, instead of one (Luan et al., 2006). These tools enable exploration of all the nuances of the spatial and temporal effects of protein deficiency, and to distinguish the processes of development (assembly/formation) versus those of maturation and maintenance. Their potential was demonstrated in our recent study into the role of exocyst genes in nephrocyte filtration function which used temperature sensitive RNAi (Wen et al., 2020). The flies were crossed and initially kept at 25°C (inactive RNAi), then embryos were collected and maintained at 29°C at which temperature the UAS-RNAi-targeting transgene, with a nephrocyte-specific driver (*Dot*-Gal4), was active for functional assays. This tightly controlled system enabled distinction between SD formation and SD maintenance, and thereby demonstrated the importance of the exocyst complex in maintaining the filtration (SD) and absorption (lacunar channels) functions in fly nephrocytes. In another study, we used temperature sensitive Gal80 combined with the ubiquitous *Tubulin* promoter to initiate knock down of *Clathrin* (*Clc*) after SD assembly, to specifically study the role of Clc in SD maintenance (Wang et al., 2021). Furthermore, by combining the fly *in vivo* platform with biochemistry applications, such as TurboID and APEX, one could identify the components that play key roles during SD assembly. TurboID is an engineered biotin ligase that uses ATP to convert biotin into biotin-AMP, which covalently labels proximal proteins (Uçkun et al., 2021; Zhang et al., 2021), while APEX is an engineered peroxidase that can convert exogenous biotin for unselective covalent labelling of proximal proteins (Lobingier et al., 2017). Either biotinylated complex can then be enriched using Streptavidin beads for subsequent analysis by mass spectrometry to achieve live-cell proteomics. These technologies enable precise spatial (cell type-specific) and temporal (developmental stage-specific) resolution.

## A Glomerular Diseases Perspective

A key characteristic shared across glomerular diseases is foot process effacement. It signifies the podocyte response to injury and is marked by loss of the interdigitating pattern of foot processes typically observed between adjacent podocytes. This process disrupts SD structural integrity, which leads to reduced SD presence and thus podocyte functional deficits. The disrupted filtration capacity in return results in proteinuria. If untreated, this process progresses to podocyte detachment and culminates in end-stage renal failure. Given the essential nature of the SD to podocyte function, it is not surprising that many of the genes described above for their roles in SD formation and maintenance have been implicated in glomerular diseases.

### Slit Diaphragm Core Components in Disease

Renal disease-associated genetic variants have been found in key components of the SD such as Nephrin (NPHS1) and Podocin (NPHS2), as well as CD2AP. *NPHS1* genetic variants are causative of congenital nephrotic syndrome of the Finnish type (Kestilä et al., 1998), in which podocytes from human embryos carrying an NPHS1 mutation displayed reduced Nephrin expression (20%) and altered (cilia-specific) α -Tubulin distribution, indicative of incomplete podocyte maturation (Vukojevic et al., 2018). *NPHS2* genetic variants have been shown to cause autosomal recessive SRNS, marked by childhood onset of proteinuria with rapid progression (Boute et al., 2000). Mutations in *CD2AP* have been found in patients with SRNS and FSGS (Gigante et al., 2009). Fly nephrocytes with *cindr* (ortholog of *CD2AP*) deficiency showed severely disrupted lacunar channels and loss of SD structural integrity. Notably, these phenotypes could be rescued by expressing human reference *CD2AP*, but not by a patient allele carrying a *CD2AP* mutation (Fu et al., 2017b), thus providing a beautiful example of patient-specific, i.e. precision disease, modeling using fly nephrocytes.

### Apical-Basal Polarity Proteins in Disease

Regarding the polarity proteins, variants in both apical and the balancing basolateral components have been implicated in glomerular diseases. On the apical side, variants in *CRB2*, a key Crumbs complex member, have been linked to SRNS (Ebarasi et al., 2015) and congenital nephrosis, Finnish type (Slavotinek et al., 2015). In addition, the Crumbs and PAR apical polarity complexes have been, directly or indirectly, linked to renal cyst disease. For example, *PALS1-interacting proteins Nephrocystin-1 (NPHP1)* and *NPHP4* have been associated with nephronophthisis (NPHP), an autosomal recessive ciliopathic childhood cystic kidney disease (Wolf and Hildebrandt, 2011). On the basolateral side, variants in *DLG5* have been linked to sporadic FSGS (Yu et al., 2016). Initial functional studies using mouse podocytes deficient for *Dlg5* (shRNA) did not show any phenotype (Yu et al., 2016), possibly due to genetic redundancy. However, recent studies in fly, which has a much more compact genome, have revealed nephrocytes deficient for *Dlg* display collapse of SD structural integrity and disrupted endocytosis (Heiden et al., 2021; Mysh and Poulton, 2021).

### Cytoskeletal Proteins in Disease

Several podocyte cytoskeletal proteins have been linked to proteinuria and glomerular disease [for a comprehensive review *see* (Blaine and Dylewski, 2020)]. Variants in *anillin* (*ANLN*), a cell cycle protein that binds F-actin, were shown to cause familial FSGS. Functional studies found that the mutated protein has reduced CD2AP binding affinity. CD2AP is the major binding partner of Endophilin and an SD-associated scaffold protein. *Anln* deficiency in mice resulted in disrupted filtration barrier integrity, foot process effacement, and severe edema (Gbadegesin et al., 2014). Mutations in *ACTN4*, which encodes an Actin-filament crosslinking protein, caused idiopathic FSGS with autosomal dominant inheritance in three families suffering from increased urinary protein excretion and decreased kidney function, with ultimate progression to end-stage renal failure (Kaplan et al., 2000). An Actin-regulating protein encoded by *inverted formin 2* (*INF2*) has been associated with FSGS. Linkage analysis and subsequent sequencing identified nine independent missense mutations in highly conserved amino acid residues across multiple unrelated families (Brown et al., 2010). Since then, at least 45 pathogenic mutations in *IFN2* have been identified in isolated FSGS (Labat-de-Hoz and Alonso, 2020). Four patient variants in *INF2* have been studied in *Drosophila* nephrocytes for *in vivo* validation. Nephrocytes expressing these variants showed a disrupted Actin cytoskeleton, in which the level of Actin accumulation in the cytosol correlated with the presumed impact of the mutation on INF2 activation. Furthermore, they showed that these *INF2* mutations were similarly correlated to reduced Sns at the cell surface, indicative of disrupted SD structures (Bayraktar et al., 2020). Further, variants in *MYH9*, which encodes an Actin-binding cytoskeleton regulatory protein, have been associated with FSGS in the African American population. Of interest, the MYH9 risk alleles were found more frequently among African Americans, whereas the protective alleles were more frequent among European Americans (Kopp et al., 2008). Another study associated variants in *MYH9* with Alport-like-syndrome (a.k.a. Fechtner syndrome; FTNS), a rare disorder with a renal component. The genetic variants were not fully penetrant for the renal phenotype, but a subset of the patients carrying *MYH9* variants suffered from nephritis, including display of foot process effacement, loss of SDs, proteinuria, and renal failure (Ghiggeri et al., 2003).

*In vitro* studies have shown that podocytes are typically in a RhoA-dependent stationary rest state. In response to stress they become migratory and CDC42 and RAC1-dependent (Mundel and Reiser, 2010). This switch might drive foot process effacement. Indeed, several Rho GTPase signaling regulators have been implicated in glomerular diseases. For example, Rho GTPase ARHGAP24 was identified as an Actin regulatory protein in podocytes, and *ARHGAP24* mutations have been found in FSGS patients and associated with reduced RAC1-GAP activity (Akilesh et al., 2011). Another example is ARHGDIA, which forms a complex with Rho GTPase. Mutations in *ARHGDIA* were found in patients with SRNS. The ARHGDIA mutations resulted in impeded interaction with RAC1 and CDC42 and increased migration of human immortalized podocytes (*in vitro*) (Gee et al., 2013). Further study found that cultured mouse immortalized podocytes carrying *Arhgdia* mutations showed RAC1 hyperactivity, as well as impaired Actin polymerization, decreased cell size, increased cellular projections, and reduced motility. This study further showed that ARHGDIA mRNA expression increased as the podocytes matured with RAC1 activity limited to immature podocytes, suggesting ARHGDIA typically suppresses RAC1 activity (Auguste et al., 2016). Note the different effect of mutant *ARHGDIA* on podocyte motility reported in each study. These conflicting results might be due to methodological differences between the two studies. Both studies used immortalized podocytes lines in which human *ARHGDIA* carrying patient mutations was overexpressed. However, they differ in that the mouse podocyte study knocked down endogenous *Arhgdia* expression (shRNA) (Auguste et al., 2016), whereas the human podocyte study did not (Gee et al., 2013). The mouse study reported trending reduced motility for three *ARHGDIA* variants based on a wound healing assay, including the two variants from the human podocyte study. However, they only provided real-time migration data for the third variant. The human podocyte study applied the real-time migration assay to both variants. Whether these methodological differences account for the opposing motility findings, or if an unknown biological process is the cause warrants further investigation. The last example is the *KANK* family genes. Recessive mutations in *KANK1*, *KANK2*, and *KANK4* have been identified in patients with SRNS. Knockdown of *kank2* in zebrafish caused a nephrotic syndrome phenotype, marked by proteinuria and podocyte foot process effacement. *Drosophila* has one homolog (*dKank*), and knockdown of *dKank* (RNAi) in flies resulted in disrupted lacunar channel and SD filtration structures. Additional assays in rat glomeruli and cultured human podocytes showed KANK2 interacts with ARHGDIA, with knockdown of *KANK2* (*in vitro*) leading to increased active (GTP-bound) RhoA and decreased podocyte motility (Gee et al., 2015).

### Endocytosis and Exocytosis Pathway Proteins in Disease

A variant in *RPH3A* has been associated with increased risk for microalbuminuria. Both in subjects with a microalbuminuric and in those with a normoalbuminuric metabolomic profile the variant was associated with urinary albumin excretion. Increased levels of albumin in urine have been associated with cardiovascular and renal disease (Marrachelli et al., 2014). Moreover, expression of the RPH3A-RAB3 complex was decreased in podocytes of a mouse model for FSGS (growth-hormone transgenic mice), and RPH3A protein expression was increased in biopsied tissue from patients with glomerular diseases, where the expression inversely correlated to the amount of urinary proteins (Rastaldi et al., 2003). Mutations in genes encoding the RAB5 (early endocytosis)-interacting proteins—GTPase-activating protein and VPS9 domain-containing protein 1 (GAPVD1) and Rabankyrin-5 (ANKFY1)—were found to cause SRNS in patients (Hermle et al., 2018). These variants were shown to reduce protein affinity for active RAB5 and an inability to rescue the migratory defect in podocytes deficient for either *GAPVD1* or *ANKFY1*. Of note, in rat glomeruli, GAPVD1 was shown to directly interact with Nephrin, a core component of the SD (Hermle et al., 2018). Mutations in the gene encoding TBC1 domain family member 8B (TBC1D8B), a regulator of RAB11 (recycling endosomes), have been identified in multiple families with SNRS (Kampf et al., 2019). Mutant TBC1D8B showed reduced affinity for RAB11 and Nephrin, and *Drosophila* nephrocytes deficient for *Tbc1d8b* showed mis-localized Sns (mammalian Nephrin) and impaired protein uptake function (Kampf et al., 2019). Additional variants in endocytosis proteins important for SD maintenance and function have been associated with nephrotic syndrome in patients, including variants in Myosin-1E (Myo1E; binds Dynamin and Synaptojanin) (Mele et al., 2011; Sanna-Cherchi et al., 2011), and CD2AP (a major binding partner of Endophilin) (Kim et al., 2003). Finally, exome sequencing revealed a mutation in *EXOC8* to segregate with disease in a family with Joubert syndrome (Dixon-Salazar et al., 2012). EXOC8 is a component of the exocyst complex, that mediates vesicle tethering for fusion in endocytosis and recycling.

## How the Fruit Fly can Contribute to the Study of Human Glomerular Diseases

### Identification and Validation of Candidate Genes for Glomerular Diseases Using the Fly System

One of the first studies using flies to model glomerular diseases, applied an unbiased genetic screen of over 1,000 RNAi transgenic lines spanning the fly genome (Zhang et al., 2013). Transgenic flies carried an ANF secretion peptide with RFP reporter driven by the myosin heavy chain enhancer (*MHC*-*ANF*-RFP), combined with RNAi (UAS-RNAi) driven by the *Hand* pericardial cell marker (*Hand*-GFP, Dot-Gal4). The secreted ANF-RFP accumulates in the nephrocytes and serves as a measure of nephrocyte function. The embryos from these RNAi crosses were rapidly assessed for RFP accumulation in the pericardial nephrocytes, with follow-up RFP up-take measurements in newly hatched adults. The screen identified over 70 genes required for nephrocyte function, all of which have highly conserved human homologs (Zhang et al., 2013). Remarkably, the RNAi lines available from *Drosophila* resource stock centres, like the Vienna Drosophila Resource Center (VDRC; Austria) and the Bloomington Drosophila Stock Center (BDSC; Indiana University, United States), collectively cover over 90% of the fly protein-coding genes. These resources will be invaluable in unbiased approaches to identify the genetic interactors of glomerular disease genes, and to gain insight in the underlying pathological molecular pathways.

The previous examples described studies that have linked genetic variants with various glomerular diseases using patient samples. Several of those studies included subsequent functional validation using *in vitro* (human) cell culture or *in vivo* animal models, such as mouse, rat, zebrafish, and flies. However, many more genes await genetic validation and subsequent mechanistic study. *Drosophila* could provide a fast and cost-effective means to achieve this, due to the highly conserved genes, pathways, and functionality between nephrocytes and podocytes. The fly can be used to rapidly survey large numbers of genes as demonstrated in an *in vivo* genetic screen of 40 genes associated with nephrotic syndrome, which validated the involvement in nephrocyte function for 34 of the genes (85%) (Fu et al., 2017b); and, in an *in vivo* RNAi screen of 29 genes with variants associated with SRNS that validated 16 genes (55%) to exert significant roles in nephrocyte function and SD structural integrity (Hermle et al., 2017). Other studies using flies have uncovered potential therapeutic targets, such as one into recessive mutations in *KANK* family members in patients with nephrotic syndrome (Gee et al., 2015). The variants were found to act through dysregulated Rho GTPase signalling, suggesting (ant)agonists of Rho GTPase activity might provide therapeutic benefit in patients with *KANK* mutations. Of note, KANK2 was shown to interact with ARHGDIA (Gee et al., 2015), a known regulator of Rho GTPases in podocytes that had been previously shown to cause SRNS (Gee et al., 2013). Once candidates have been validated in fly and their initial function has been established, they can be moved forward to the more expensive and time-consuming studies in mammalian model systems. Furthermore, for various existing fly models for glomerular disease the effect of their genetic variants on nephrocyte uptake function and the structural integrity of the SD has not yet been studied. Such as the fly model to study the APOL1 risk alleles associated with glomerular diseases in persons of recent African ancestry. These flies show biphasic age-related changes in nephrocyte uptake function (Fu et al., 2017a). Of note, the effect of APOL1 risk alleles on nephrocyte function has not yet been correlated to structural changes in the SD. These studies could reveal additional mechanism regulating SD assembly and maintenance, and provide insight into the interaction of known pathways.

### Fly to Delineate the Genetic Intricacies Underlying Glomerular Diseases

Glomerular diseases caused by defective SDs typically share proteinuria and renal failure in their clinical profile. However, the shared structural cause, a defective SD, is the culmination of varying underlying disease mechanisms. Glomerular disease modelling is further complicated by variants in a single gene leading to different outcomes. For example, variants in *INF2* have been associated with both FSGS and Charcot-Marie-Tooth disease, a neurological disorder with nephrotic pathology (typically FSGS). One study screened over 50 autosomal dominant *INF2* mutants from patients in HeLa *INF2* KO cells, and further assessed a few variants in *Drosophila* nephrocytes and one in patient urine-derived epithelial cells (Bayraktar et al., 2020). The fly data demonstrated intracellular accumulation of Actin in nephrocytes carrying an *INF2* patient variant. Furthermore, the Actin accumulation quantitatively correlated with disrupted localization of Nephrin to the plasma membrane. Together the findings enabled a clear distinction between those variants causing primary FSGS and those that cause FSGS with the neurological disorder, as well as the definition of subsets of *INF2* variants based on their level of activation and intracellular Actin accumulation (Bayraktar et al., 2020). Furthermore, in other cases the genetic cause of glomerular disease lies in multiple genes (polygenic). For example, using comprehensive modelling in flies, variants in *Adducin-* γ [*ADD3*; *hu li tai shao* (*hts*) in fly] and *Lysine Acetyltransferase 2B* (*KAT2B*; *Gcn5* in fly) were shown to act synergistically in kidney and heart dysfunction comorbid with *ADD3*-associated phenotypes (Gonçalves et al., 2018). Or in the case of *MYH9* where the same genetic variant resulted in diverse phenotypic outcomes (all carriers displayed platelet and leukocyte abnormalities, but only a subset suffered from proteinuria and renal failure among other symptoms), suggesting that additional genetic and/or environmental factors contribute to disease outcomes (Ghiggeri et al., 2003). Technical limitations and time constrictions make modeling in mice unfeasible for these applications. The fly on the other hand allows for fast screening of multiple variants for various phenotypic outcomes, and new lines can be rapidly generated to carry patient (-specific) variants in multiple genes.

### Drosophila Enables Rapid, Scale-Able, *in vivo* Drug Screens

Several studies have used *Drosophila* nephrocytes to complement a mammalian model system. One such study used a combination of *in vitro* podocyte and *ex vivo* nephrocyte models to investigate the phosphorylation of SD proteins Nephrin and NEPH1 in maintenance of the Actin cytoskeleton (Solanki et al., 2021). Fly nephrocytes were treated with hepatocyte growth factor (HGF) following chemically induced injury with protamine sulfate, which resulted in severe Actin cytoskeletal disorganization. They demonstrated HGF-induced phosphorylation of Sns (mammalian Nephrin) and Duf (mammalian NEPH1) mediated nephrocyte recovery. The same response was observed when treating immortalized human podocytes *in vitro*, thus exemplifying the molecular conservation between nephrocytes and podocytes and the potential to use nephrocytes in drug screens. Another study identified variants in *aarF domain containing kinase 4* (*ADCK4*) in multiple patients with familial SRNS (Ashraf et al., 2013). They used flies and zebrafish for functional validation and demonstrated phenotypic attenuation by dietary supplementation with CoQ10. *ADCK4* encodes a kinase that acts in the biosynthesis of coenzyme Q (ubiquinone); while not a direct SD component, a later study in *Drosophila* showed CoQ10 pathway gene deficiency leads to abnormal localization of SDs, collapse of lacunar channels, and dysmorphic mitochondria, as well as increased autophagy and mitophagy, ROS, and sensitivity to oxidative stress (Zhu et al., 2017c). Similarly, these effects in fly nephrocytes could be attenuated by dietary supplementation with CoQ10. Moreover, the latter study showed that expressing the human *COQ2* reference gene was able to attenuate the protein uptake defect in *Coq2*-deficient nephrocytes, whereas expressing a patient allele carrying a *COQ2* variant could not (Zhu et al., 2017c). This study is notable for demonstrating the possibility of using *Drosophila* to generate precision disease models, designed to study patient-specific mutations and underlying mechanisms, and to identify therapeutic targets and screen compounds for their effectivity in reversing the phenotypes. These genetic and pharmacological *in vivo* screens in *Drosophila* can be readily scaled up to comprise well over 100 genes as we have shown in studies into candidate genes for congenital heart disease (CHD) to demonstrate their importance in nephrocyte function (Zhu et al., 2017b; 2017a). This study also provided functional validation for the CHD patient-derived *WDR5* ^*K7Q*^ allele. Replacing endogenous *wds* expression with the patient allele could not restore the cardiac phenotype induced by *wds* deficiency, whereas the human reference allele could (Zhu et al., 2017a). In a recent a study, we used transgenic flies carrying the human *KRAS* ^*G12V*^ leukemia-variant in both genetic and chemical inhibitor screens, which independently revealed the importance of hypoxia signalling in mediating RAS-induced cancer phenotypes (Zhu et al., 2022). Together these studies show how fly can help in studying human glomerular diseases, for both functional validation and precision disease modelling, as well as for therapeutic drug screens.

## Concluding Remarks

The genetic, molecular, structural, as well as the genetic deficiency-induced phenotypic similarities, make the *Drosophila* nephrocyte a well-suited model to study the SD in development and disease. Especially the many genetic tools that enable defined temporal and spatial control put the fly in a strong position to study the intricacies of SD formation vs. maintenance. Moreover, the fly system is ideally equipped for rapid, extensive genetic and pharmacological *in vivo* screens, and can be used to study the interplay of multiple components at the SD. Finally, the fly provides a powerful system to take glomerular disease studies into the future of precision disease modeling to identify and tailor therapeutics to the individual patient.
